# A Survey of Knowledge, Attitude and Practice of Nurses towards Pharamacovigilance in Taleqani Hospital

**Published:** 2010

**Authors:** Giti Hajebi, Seyed Alireza Mortazavi, Jamshid Salamzadeh, Aed Zian

**Affiliations:** *School of Pharmacy, Shaheed Beheshti University of Medical Sciences, Tehran, Iran.*

**Keywords:** Adverse drug reaction, Knowledge, Attitude, Practice, Nurses, Educational program, Voluntary reporting of ADRs

## Abstract

Detection of probable harmful consequences arised from the usage of pharmaceutical products requires decisive, continuous and close monitoring by medical staff whom should have knowledge of adverse drug reactions and they should also have to report any suspected instances, when any kind of adverse drug reactions have been observed. This study has been carried out on the knowledge, attitude and practice of nurses towards pharmacovigilance in the Taleqani medical, teaching and treatment center in Tehran, before and after an ADR educational program. This study was commenced in March 2005 and ended in October 2005, using a questionnaire through two steps. In every step, 150 questionnaires were distributed in various wards of the Taleqani Hospital. Collected data were entered into the Excel software and then data analyzed using the SPSS statistical software. Familiarity of nurses with the ADR center and it’s duties is the first step in training how to report ADR, and could help to enhance thire awareness. The use of lecture training for increasing the awareness of nurses was found to be very effective. Regression multivariable analyses showed that the knowledge of nurses, regarding previous familiarity with the ADR center is better than the others (r = 0.38, P = 0.01), and the attitude of female nurses is better than males (r = 0.27, P = 0.01). According to the statistical results, the knowledge of nurses before the seminar was significantly less than the knowledge after the seminar (P= 0.0001), but there was no significant effect on the attitude (P= 0.05). Regarding the submission place of ADR reports, only 3.4% of nurses pointed out to the ADR center. Before and after training, a limited duration of time was reported to be the most restricted factor for clinical recognition of adverse drugs. Based on the results of this study, it is necessary to offer continuous ADR educational program until we reach the point that voluntary reporting of adverse drug reactions becomes conventional and habitual among the nursing staff.

## Introduction

Adverse drug reactions (ADRs) are known as very important causes for hospitalization (1). ADRs occur approximately in 30% of hospitalized patients, and patients in the ICU wards are exposed to more danger than the others (2). ADR can be a threat for patient’s safety and quality of their life and may impose a lot of costs on the health systems (3). The important point about ADRs is pharmacovigilance or the methods used for their recording, evaluation and prevention (4). Unfortunately, in Iran not enough attention has been paied to this matter. In USA, more than one hundred thousand death-induced ADRs occur yearly, and also 7% of hospitalizations are related to ADRs (3, 5).

ADR is the fourth cause of death in USA. In 1994 the cost due to ADRs was 4 billion dollars (6). On the other hand, in a published report by FDA in 1989, 12000 cases of death were due to ADRs (7). Nurses have a unique position to monitor patients drug therapy, however, voluntary ADRs reporting, based on monitoring safety of medicine, must be a major responsibility for all the health care professionals (8). Knowledge and awareness of nurses with respect to the effects, adverse effects and methods of administration of drug, could help to elevate the quality of pharmacotherapy in hospitals. Reporting system and sending yellow cards to drug safety committee was innovated in 1964. The yellow card includes three parts: Information about the patient, suspected drugs causing ADRs and information about the submission date and the reporter (9). It is not possible to prevent every ADR, but the knowledge of nurses is very effective to decrease the rate of occurrence of ADRs (10, 11). Considering the results of several studies (12-14), the positive effect of education on the elevation of knowledge, improvement of attitude and practice, as well as the promotion of quality of nursing care could be observed. However, no such studies have been conducted in Iran. This study has been carried out on the knowledge, attitude and practice of nurses towards pharmacovigilance in Taleghani Medical, teaching and treatment center in Tehran, before and after an ADR educational program in the form of a seminar and hand-outs.

## Experimental

This semi-experimental study was conducted in the Taleghani Hospital from March 2005 to October 2005, and 250 nurses and nursing aid staff (male and female) participated in this study. First, a questionnaire in three parts was constructed. It included the following sections:

1. Demographic information of participants and questions about their familiarity with the ADR center.

2. Nineteen questions about the knowledge of nurses regarding drugs and their adverse effects.

3. Seventeen questions related to the attitude of nurses towards pharmacovigilance.

After obtaining permission from the hospital authorities, questionnaires were distributed in all the hospital wards, through two steps. 

In the first step, the extent of knowledge and attitude of nurses towards pharmacovigilance before the educational program were collected using the questionnaire. Three months after conducting the first step, nurses participated in an educational program held by the Iranian ADR center. In addition, hand-outs on ADR and means of its reporting were distributed in all the wards. Two months after the educational program, the nurses were re-evaluated by the same questionnaire.

## Results and Disscussion

In the first step, 90 filled questionnaires were obtained (74 women and 10 men). Six forms were omitted from the study, due to being uncompleted.

In the second step, 71 completed forms were obtained (59 women and 12 men).

Twelve nursing staff participated in the seminar (10 women and 2 men).The age of the nurses were between 20 and 54, and in terms of education, most participants had a bachelor’s degree.

Talegheni Medical center has emergency, internal, women, obstetrics, radiology, ENT, hematology, orthopedics, dialysis, ICU, general surgery, heart, endocrine, digestion, psychology, NICU, CCU, vascular surgery and pediatrics wards. 

The collected data were entered into the Excel software, and then analyzed with the SPSS statistical software. Following the preliminary calculations, data were statistically analyzed and for the assimilation of data, first of all based on the type of variation, appropriate tests were selected for the preliminary analysis.

In order to evaluate the relationship between these variations with knowledge and attitude, a level of significance of p ≤ 0.2 was used. Those variations with a p-value of less and equal to 0.2, entered the regression multi-variable analyzes (the Ridge regression).

Regression multi-variable analyzes showed that the knowledge of nurses, regarding previous familiarity with the ADR center, is better than the others (p = 0.01), and the attitude of female nurses is better than males (p = 0.01). According to the statistical results, the knowledge of nurses before the seminar was significantly less than the knowledge after the seminar (p = 0.0001), but there was no significant effect on the attitude (p = 0.05). Determination and definition of variables has been shown in Table1. 

**Table 1:** Determination and definition of variables

**Variable **	**Variable type **	**Definition **
Age	Independent/Quantitative	Years
Sex	Independent/ Qualitative/ Nominal	Male or female
Education	Independent/ Qualitative/ Nominal	High- school diploma; higher diploma; Bachelor; Above Bachelor
Ward	Independent/ Qualitative/ Nominal	Wards were divided into 4 main groups
Professional experience	Independent/Quantitative	Number of employment years
Familiar with the ADR center	Independent/ Qualitative/ Nominal	Familiar or not familiar with the ADR center
Knowledge of nurses	Dependent/Ratio	Correct answers to the questions in the questionnaire
Attitude of nurses	Dependent/Ratio	Marks gained from the questions on attitude evaluation

Table 2 presents the relationship between knowledge and variables (before training). In Table 3, the relationship between attitude and variables (before training) has been shown.

**Table 2:** Relationship between the knowledge and variables (before training)

**Independent variables **	**Preliminary statistical test **	**Results **	**Relationship **
Age	Rank correlation	p = 0.19	N.S.*
Sex	Mann-Whitney	p > 0.2	N.S.*
Professional experience	Rank correlation	p > 0.2	N.S.*
Education	Mann-Whitney	p > 0.2	N.S.*
Ward	Kruskall-Wallis	p = 0.001	Nurses in different wards had different knowledge
Familiar with the ADR center	Mann-Whitney	p = 0.001	Staff familiar with the ADR center had a higher knowledge

*N.S.= a non-significant statistical relationship

**Table 3:** Relationship between the attitude and variables (before training).

**Independent variables **	**Preliminary statistical test **	**Results **	**Relationship **
Age	Rank correlation	p = 0.07	N.S.*
Sex	Mann-Whitney	p = 0.04	Attitude of female nurses was better than the male nurses
Professional experience	Rank correlation	p = 0.16	N.S.*
Education	Mann-Whitney	p > 0.2	N.S.*
Ward	Kruskall-Wallis	p = 0.001	Nurses in different wards had different attitudes
Familiar with the ADR center	Mann-Whitney	p > 0.2	N.S.*

*N.S.= a non-significant statistical relationship

The relationship between knowledge and variables (after training) has been shown in Table 4. 

**Table 4:** Relationship between the knowledge and variables (after training).

**Independent variables **	**Preliminary statistical test **	**Results **	**Relationship **
Age	Rank correlation	p = 0.19	N.S.*
Sex	T-test	p = 0.18	N.S.*
Professional experience	Rank correlation	p = 0.34	N.S.*
Education	T-test	p = 0.29	N.S.*
Ward	ANOVA	p = 0.7	N.S.*
Familiar with the ADR centre	T-test	p = 0.04	Staff familiar with the ADR centre had a higher knowledge
Participants in the seminar	T-test	p = 0.18	N.S.*
Course hand-outs	T-test	p = 0.23	N.S.*

*N.S.= a non-significant statistical relationship

**Figure 1:**
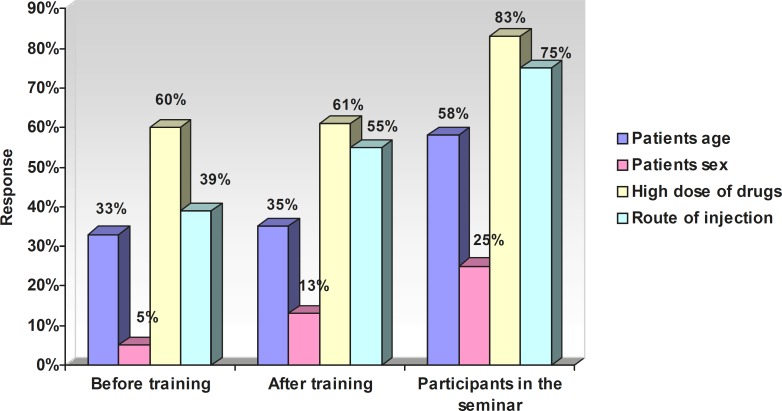
The response of nurses (percentage) towards the risk factors of ADRs

**Figure 2:**
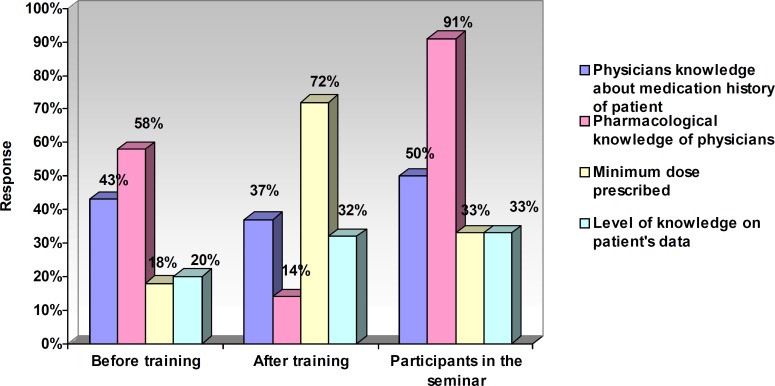
The response of nurses (percentage) towards the factors related to a reduced rate of ADRs.

Table 5 presents the relationship between attitude and variables (after training). Figure 1 represents the response (percentage) of nurses towards the risk factors of ADRs. The response of nurses (percentage) towards factors related to a reduced rate of ADRs has been shown in Figure 2. 

**Table 5:** Relationship between the attitude and variables (after training). All the results were found to be non- significant

**Independent variables**	**Preliminary statistical test**	**Results**
Age	Rank correlation	p > 0.2
Sex	Mann-Whitney	p > 0.2
Professional experience	Rank correlation	p > 0.2
Education	Mann-Whitney	p = 0.14
Ward	Kruskall-Wallis	p = 0.10
Familiar with the ADR center	Mann-Whitney	p = 0.49
Participants in the seminar	Mann-Whitney	p > 0.2
Course hand-outs	Mann-Whitney	p > 0.2

*The relationship between knowledge and possible variations*

Nurses familiar with the ADR center had more awareness than the others. This finding is quite logical and shows that this center is effective. 

Before training, 48% of respondents knew the ADR center. After training this figure raised to 50%. The extent of respondents who participated in seminar was 91%. It was found that educating via the lecture method (seminar) was more effective than educating via booklets. The results of this study about familiarizing the participants with the ADR center is in agreement with the results obtained in the Rhode Island hospital study (15). 

Familiarity of nurses with the ADR center and its duties is the first step in training how to report ADR, and could help to enhance their awareness. Similar results were obtained from a study conducted in the pharmacy ward of South Mead hospital in England (9). 

Another study in China in 2004, showed that the lack of basic knowledge on ADR and the voluntary reporting system, are the main reasons for under-reporting (12). 

*The relationship between attitude and possible variations*

Based on this study, being merely familiar with the ADR center has a direct relationship with the attitude of nurses. However, the two different training methods had no extensive and clear influence on their attitude. 

Our training resulted in an increased rate of familiarity among nurses, towards the ADR center. It seems that for improving the attitude, continuous training is needed. 

The results of this study show that female respondents have a better attitude than male respondents. Unfortunately, due to the lack of any similar previous study, it is not possible to compare the results. 

*Kowledge of nurses*

Talidomide occurrence in 1961 triggered the formation of national ADR centers for registering and analyzing side effects of drugs in different countries and this itself led to the start of ADR voluntary reporting system. 

Only 32% of our respondents knew this matter, and following booklet training, the results did not change, but after the seminar training it enhanced to 75%. Scarce knowledge about this could be related to the deficiency of information on ADR, and how to construct a reporting system. The use of lecture training for increasing the awareness of nurses was found to be very effective. 45% of respondents stated that WHO is now leading the voluntary ADRs reporting system. After booklet and lecture training, this value increased to 55% and 83%, respectively. In fact worldwide voluntary ADR reports by WHO have shown their utter importance and awareness by WHO has resulted in a greater attention of health-care professionals towards its importance. This is not possible, unless through continuous training. Regarding this matter, our education (both methods) was found to be effective. 

Around 10% of drugs, after entering the market, are recalled due to ADRs. Knowing this issue by nurses can enhance their motivation to report ADRs and holding out seminars could help to inform them on this matter. 

68% of nurses stated that the ADR center is in contact with the health-care professionals via newsletter, scientific posters and various lectures. 

44% of nurses agreed that the recognition of ADRs does not need a large amount of samples. By continuous training, the number and rate of reports can increase, until no ADR is missed.

In terms of the definition of ADR, 75% of our respondents answered correctly, while in a similar study in China this rate was only 1.6% (12).

A correct concept on ADR is the first step towards improving the way to register and report them.

The awareness of our respondents towards the necessity of reporting side effects induced by herbal, chemical, blood products, vaccines, contrast media and dental drugs, was at a high level and after training it became even better than before. Indeed, ADR reporting is not restricted to a special drug. 

About 75% of ADRs depend on the dose, but most of our nurses proclaimed less than 50%. Through holding continuous training programs, dependence of ADRs on the dose of drug could be further emphasized.

Before and after training, a limited duration of time was reported to be the most restricting factor for clinical recognition of adverse drugs. A similar study also detected a limited time as the reason for under-reporting (16). 

Our training managed to increase the awareness of nurses towards the risk factors for the occurrence of ADRs. 60% of them being due to high dose, 5% due to sex, 23% due to high age and 39% due to the route of administration. 

Education was found to be very effective in enhancing the level of awareness of respondents towards pharmacovigilance and its goals, since the rate raised from 24% before training to 31% and 42%, respectively, after booklet and lecture training. 

*Attitude of nurses*

In general, the attitude of our nurses towards ADRs and its reporting was found to be acceptable, and both the training methods adopted in this study caused little improvement on it. 91% of the respondents propounded that ADR reporting is one of the duties of health-care professionals, while 69% stated that ADR reporting is specifically the duty of pharmacists. This is an obvious paradox, since all the health-care professionals have responsibilities on this matter. 86% of nurses agreed to commence the ADR reporting system in the hospital. This finding seems to be logical and in many countries had shown good results.

In 1999, in South Mead hospital in England, a center for evaluation and adjustment of ADR reports sat in motion and after ten months the number of reports enhanced extensively (9).

Existence of ADR center in hospital can increase the awareness of physicians, nurses and pharmacists towards ADRs and the number of ADR reports.

Despite the fundamental importance of reporting of suspected adverse drug reactions, less than 10 % of serious adverse drug reactions are reported (17).

ADR reporting does not need to confirm that there is a relationship between a drug and side effect, but if there is a doubt it can be reported. Every ADR should be reported, even if it is not well–known.

In a study on physicians, 28% of them did not report ADR, because of a lack of confidence on the reason for that particular ADR (18).

In our study, 44% of respondents believed that ADR reporting is the duty of pharmaceutical companies and legal medical authorities. This opinion is basicly wrong. like we said before it is the duty of all heath care professionals. 60% of nurses believed that ADR reporting can cause legal challenges. This wrong belief is one of the main reasons for the lack of ADR reporting. Through suitable training, this wrong belief could be changed.

Regarding the question that due to a poor quality of Iranian drugs, ADR reporting is worthless, 77% of respondents were against it, and this justified the reporting of Iranian drug ADRs. 50% of respondents believed that ADR reporting can cause omission of drugs and result in limitation in physicians drug selection. However, this is not true, since there are many alternative drugs. 73% of nurses addressed that side effects of drugs in Iran are not less than the advanced countries. This matter shows that they have witnessed many ADRs, but inspite of that, ADR reporting in Iran is scarce. Only 52% of participants believed that mortality induced ADR is an inseparable part of therapeutic processes. 77% of respondents proclaimed that ADR repoting can help to determine the quality of drug products. This finding complies with a study in Sweden, stating that “ADR reporting by nurses could improve the overall safety of drugs” (13).

The opinion of 68% of nurses in our study was that all ADRs are valuable and should be reported, specially severe and life threatening cases. In a study in China, more than 80% of nurses stated that dangerous and rare ADRs as well as side effects of new drugs should be reported (12). In another study in Germany in 2002, knowledge and attitude of physicians towards ADRs was investigated. 70 % of respondents believed that observing ADRs raises no concern and there is no need to report them (19). 

*ADR reporting and sending place*

92% of our respondents, when encountered with an ADR, made an effort to report it and only 30% of them stated that they had encountered with an ADR before. In a similar study in China (12), 85% of nurses had encountered with an ADR before, but only 22% of them made an effort to report them. In comparison, our respondents had a greater awareness than Chinese nurses towards the importance of ADR reporting.

By analyzing the answers of our nurses, we found that 70% of them had never encountered an ADR. This rate also included doubtful cases. Regarding the high occurrence of ADR, these results show the unability of nurses to recognize ADRs.

About the sending place of ADR reports, only 3.4% of nurses pointed out to the ADR center and this rate in the study in China was 2.9% (12). As could be seen, the reporting trends in these two countries are similar.

Although the nurses knew the obligation of ADR reporting, but only a low percentage of them knew that the ADR center is a place for sending the reports. It was found that educating via lecture (seminar) was more effective than educating via booklets.

Most nurses used to send their reports to physicians in the ward (56%), headnurse (26%), and pharmacy (13%). In the study in China, respondents stated, in order, hospital pharmacies, pharmaceutical companies and drug centers within the province, as the main places for reporting ADRs.

The answer of nurses in our study showed that the awareness of voluntary ADR reporting system is not desirable. Hence, training is necessary, until ADR reporting among them turns into a routine habit.

Based on the ADR center reports on the practice of our nurses, as from two years before the start of this study no report had been sent to the ADR center, and since the completion of this study, only one report was sent to the ADR center by our nurses.

In conclusion, it is necessary to offer continuous ADR-related educational programs until we reach the point that voluntary reporting of adverse drug reactions become conventional and habitual among the nursing staff.
